# Supplementary figures and images for: The C. elegans GATA transcription factor elt-2 mediates distinct transcriptional responses and opposite infection outcomes towards different Bacillus thuringiensis strains
Source: PLoS Pathog. 2020 Sep 24;16(9):e1008826. doi: 10.1371/journal.ppat.1008826 (PMC7513999; doi:10.1371/journal.ppat.1008826)

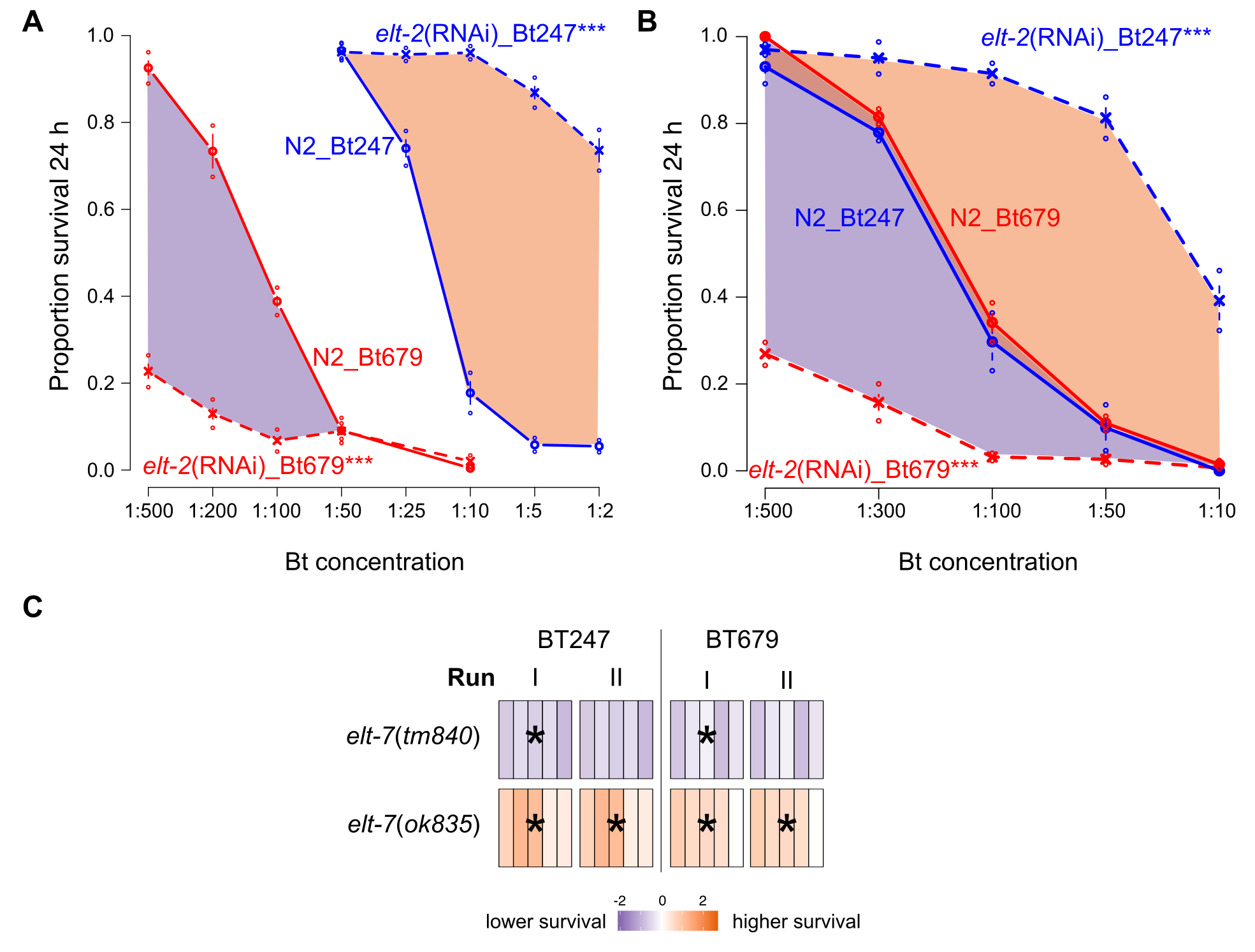

Supplement: S1 Fig — Related to Fig 3. (A) Survival of elt-2(RNAi) (dashed lines) and RNAi control worms (solid lines) 24 h p.i. with Bt247 and Bt679. The figure represents the same data that are shown in Fig 3C and 3D and in run I of Fig 3E but here they are plotted in the same coordinate axes. In this way it is visible that Bt679 is able to kill the host to the same extent as Bt247 but at lower concentration, showing that Bt679 is more virulent than Bt247. (B) Survival of elt-2(RNAi) and RNAi control worms 24 h p.i. with different cultures of Bt247 and Bt679 compared to (A). Both Bt strains kill the same proportion of hosts at the same concentration, showing that they have the same level of virulence. The opposite survival phenotype is also observed in elt-2(RNAi) worms when they are exposed to Bt247 and Bt679 with the same virulence levels. Mean and SEM are shown, N = 5 plates with 30 worms each. Statistics as in Fig 3E. *** shows p-value < 0.001 comparing RNAi to controls. Bonferroni adjusted. (C) elt-7 knockout mutants do not exhibit a Bt strain-specific survival phenotype. Heat maps show difference in survival at 24 h p.i. between wildtype N2 and elt-7(tm840) and elt-7(ok835) mutant animals. As in Fig 3, the shadowing between survival curves of elt-2(RNAi) and controls in (A-B) reflects higher or lower survival of treatment compared to control as indicated in the color scale bar of (C). (TIF) [file ppat.1008826.s001.tif]

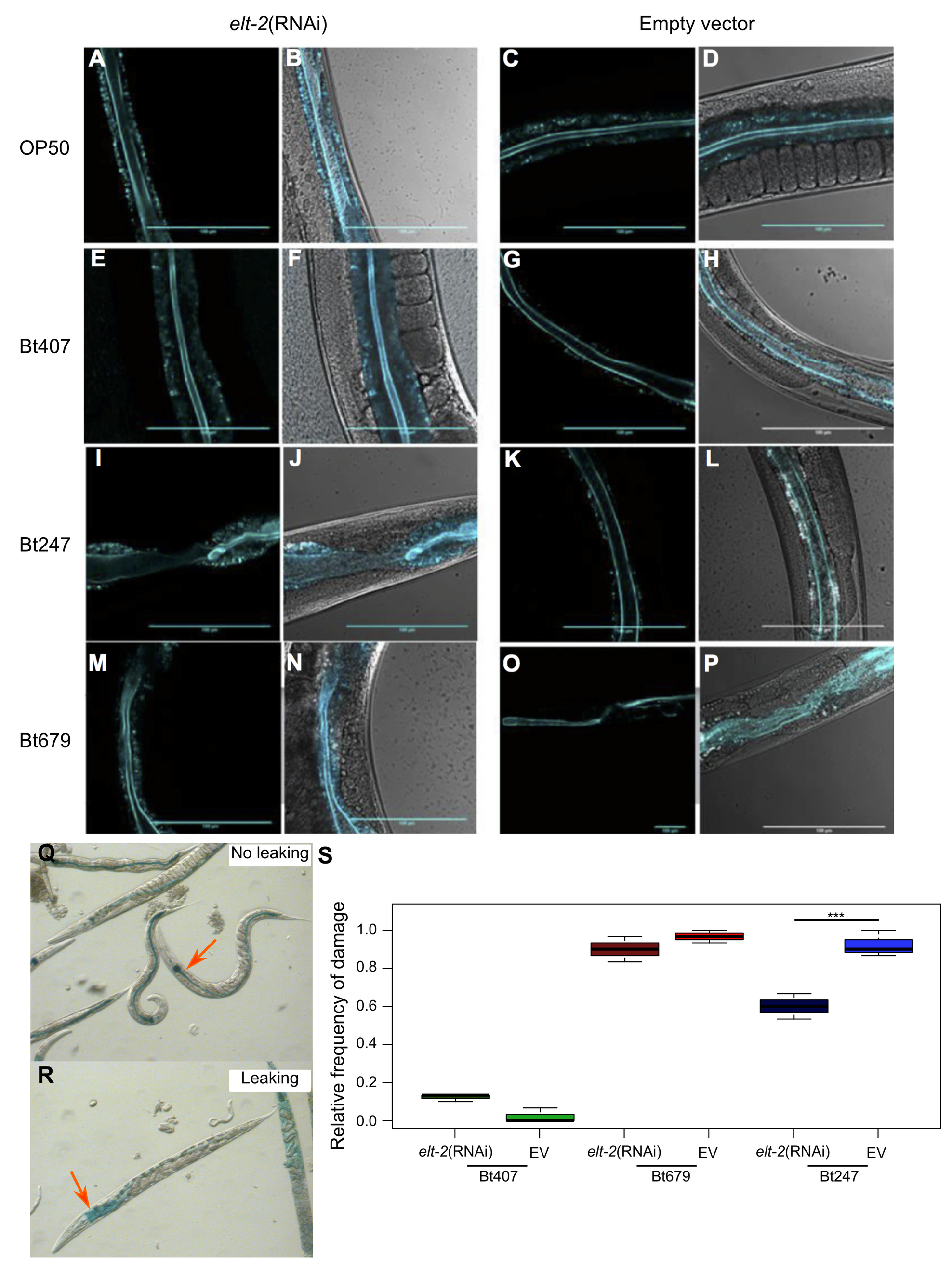

Supplement: S2 Fig — Related to Fig 4. (A-P) Fluorescence images and fluorescence merged with bright field images of the strain BJ49 carrying an IFB-2::CFP transgene 24 h after exposure to (A-D) E. coli OP50 (E-H), Bt407 (I-L) Bt247, and (M-P) Bt679. Scale bars represent 100 μm. IFB-2 is a structural component of the intestinal terminal web [99]. The CFP tag surrounds the intestinal lumen and is seen as two parallel thin continuous lines when healthy worms are inspected under the fluorescence microscope [99]. Damage in the intestinal epithelium can be observed when these continuous thin lines produce gaps, twists, blur or collapse into one line [100]. Consistent with the results of the TEM analysis (Fig 4C–4N), we found that low survival rate of elt-2(RNAi) worms after Bt679 infection coincides with exacerbated damage to the intestinal brush border and terminal web (S2O and S2P Fig) compared to empty vector controls (S2M and S2N Fig). However, higher tolerance of elt-2(RNAi) to Bt247 (S2I and S2J Fig) infection coincides with substantially less damage at the intestinal epithelium compared to control worms (S2K and S2L Fig). Worms exposed to non-pathogenic bacteria show no damage to their intestinal brush border and terminal web at 2 h p.i., regardless of the elt-2(RNAi) treatment (S2A–S2H Fig). (Q-S) Smurf assay to assess intestinal epithelium integrity [95]. At 24 h p.i. worms were exposed for 3 hours to E. coli OP50 with blue food dye. We observed if there was leaking of the dye from the intestinal lumen into the intestinal cells as a proxy for disruption of intestinal integrity. We show a representative picture of (Q) a non-leaking intestine (integral), (R) a leaking intestine (loss of integrity or damage), respectively indicated by arrows, and (S) the relative frequency of intestinal damage of worms exposed to Bt679, Bt247 and bacterial controls. Damage was scored as a binary observation (present/absent) of blue food dye leaking inside intestinal cells as opposed to remaining contai [file ppat.1008826.s002.tif]

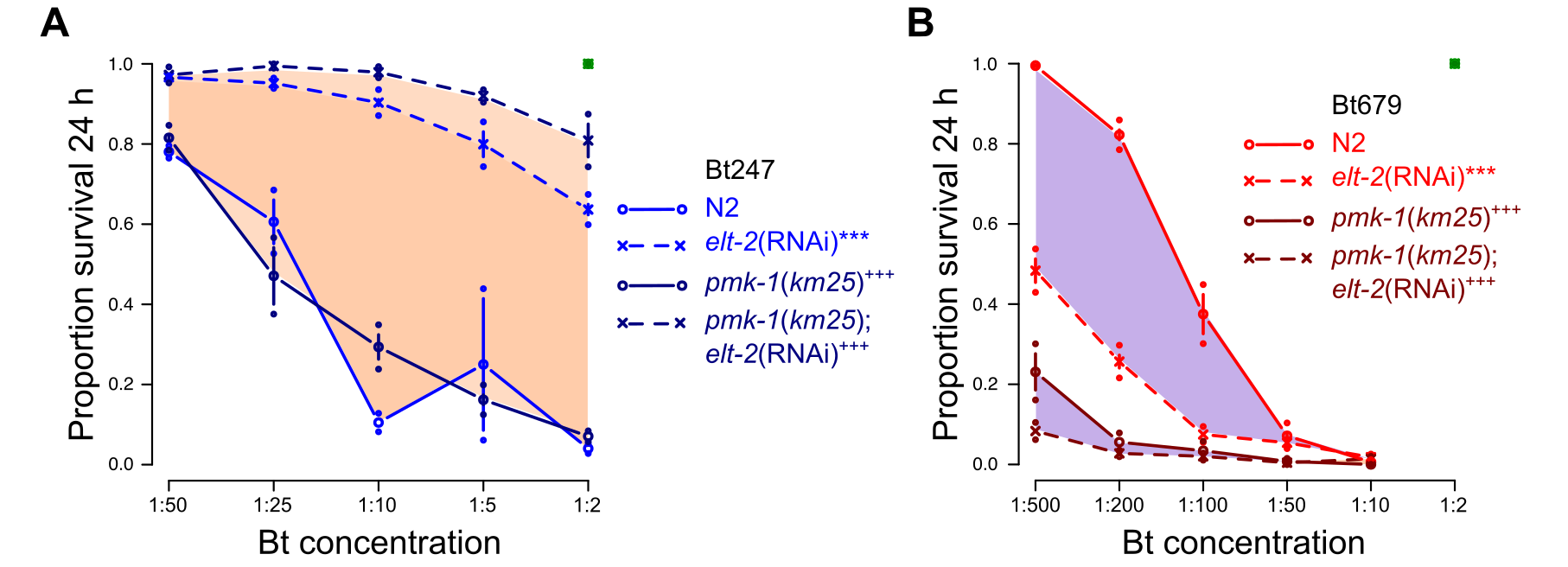

Supplement: S3 Fig — Related to Fig 5. (A and B) Survival of wildtype N2, pmk-1(km25) mutant, elt-2(RNAi), and pmk-1(km25);elt-2(RNAi) animals 24 h p.i. with (A) Bt679 and (B) Bt247. In A and B dashed lines represent worms with elt-2(RNAi) treatment. Mean and SEM are shown, N = 5 plates with 30 worms each. The figure represents the same data that are shown in Fig 5A run I. Statistics as in Fig 3E. *** shows p-value < 0.001 comparing RNAi to empty vector, +++ shows p-value < 0.001 comparing mutant to N2. Bonferroni adjusted. Green dots in (A-B) represent survival of worms exposed to Bt407. Our results suggest that the p38 MAPK pathway is required for the C. elegans defense to Bt679, but dispensable for the defense response to Bt247. (TIF) [file ppat.1008826.s003.tif]

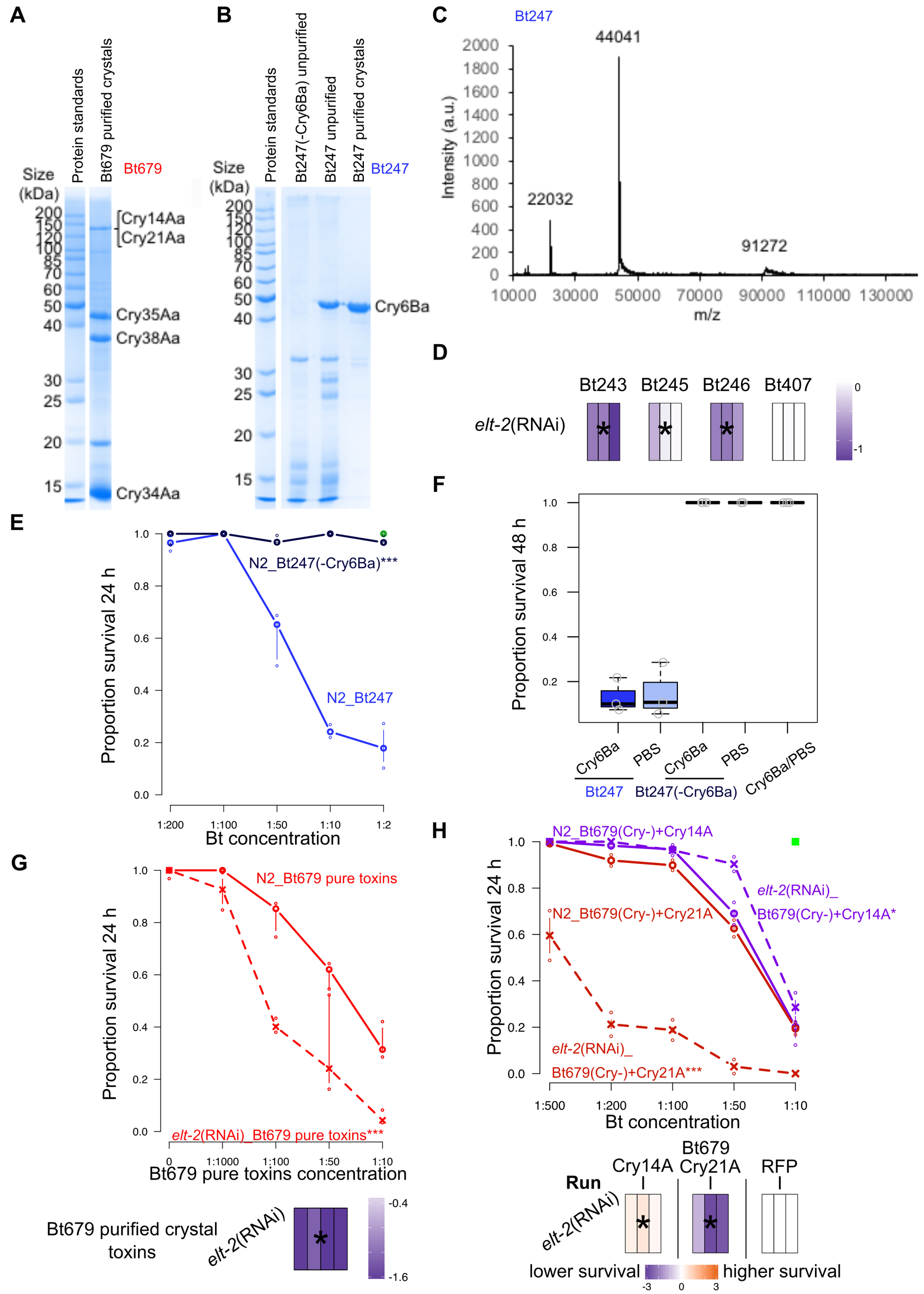

Supplement: S4 Fig — Related to Fig 5. SDS-PAGE gel (12% of samples loaded with Laemmli buffer and DTT, heated five minutes at 95°C, run at 110V for 2 h, and stained with InstantBlue overnight) showing (A) purified crystal bands from a culture of Bt679, including the nematicidal Cry toxins Cry14Aa and Cry21Aa, and (B) purified Bt247 toxin, showing one unique purified crystal band between 40 and 50 kDa (expected from sequence: 44 kDa) corresponding to Cry6Ba (not present in Bt247 -Cry6Ba). (C) MALDI analysis of the Bt247 toxin, with 34 μM (1,5mg/mL) of sample re-suspended in 100 μI water (70:30) and matrix SA dd (200 mg/mL; I/H20/TFA 70:30:0,1), confirms that the major crystal produced is a monomer of 44 kDa (expected for Cry6Ba). There are also different charges for the same toxin (bicharged = 44 kDa/ 2 charges = 22). Similarly, a small amount of dimer is detected (91 kDa). (D) elt-2(RNAi) worms are more susceptible to infection with the BT strains Bt243, Bt245, and Bt246. Difference in survival between N2 control and elt-2(RNAi) animals 24 h p.i. with additional nematicidal strains Bt243, Bt245 and Bt246. (E) Survival of N2 wildtype control animals 24 h p.i. with either wildtype Bt247, expressing the Cry6Ba toxin, or Bt247(-Cry6Ba), which lacks the toxin genes and was thus unable to kill C. elegans. (F) Boxplots show survival of N2 wildtype control animals 48 h p.i. with purified Cry6Ba toxin resuspended in PBS or in spore solutions of the indicated Bt strains. Purified Cry6Ba diluted with PBS or mixed together with Bt spores at a range of concentrations between 1:2 to 1:1000000 was not able to kill worms. (G) difference in survival between N2 wildtype controls and elt-2(RNAi) animals 24 h after exposure to different dilutions of a mix of crystal toxins purified from a culture of Bt679. (H) elt-2 is required for survival after exposure to the Bt679 PFT Cry21Aa3 but not Cry14Aa2. elt-2(RNAi) worms exposed to Bt679(-Cry) expressing either Cry14Aa2 (Cry14A), Cry21Aa3 (Cry21A) or RFP. Evol [file ppat.1008826.s004.tif]

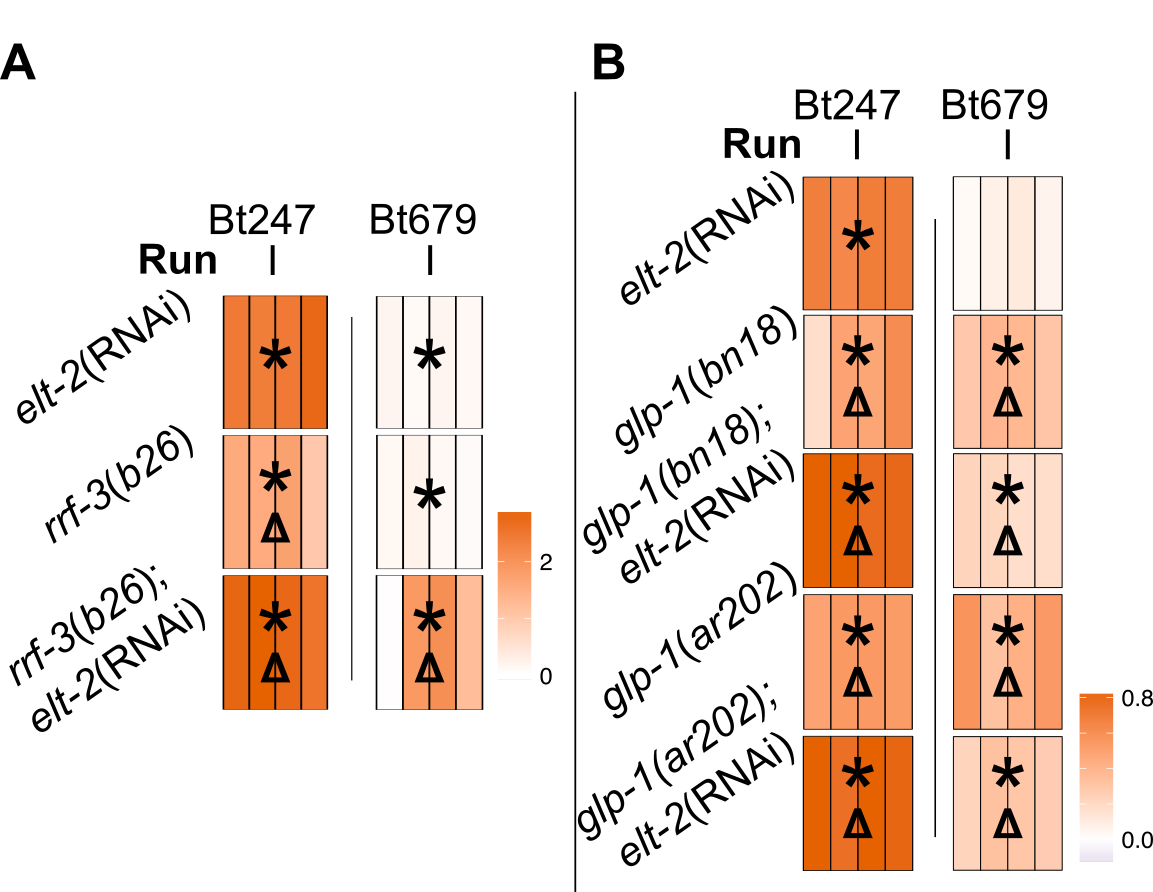

Supplement: S5 Fig — Related to Fig 6. In (A, B) we show the difference in survival between the N2 control and (A) the sperm-deficient and sterile RNA-dependent RNA polymerase rrf-3(b26) mutant; (B) the LIN-12/Notch family of receptors member glp-1(ar202) and glp-1(bn18) sterile mutants. Triangles indicate significant difference compared to elt-2(RNAi). Sterile mutants had higher survival than controls both on Bt247 and Bt679, suggesting that the effect of sterility in our infections is not Bt strain specific. Knockdown of elt-2 in the rrf-3(b26), glp-1(ar202), and glp-1(bn18) mutant backgrounds further enhanced the increased survival rate observed in sterile mutants infected with Bt247 and produced higher survival rate on Bt679 than that of elt-2(RNAi) worms alone. Our results suggest that the increased survival rate of sterile mutants on Bt247 and Bt679 is independent of the effect of elt-2. The data represented in the heatmap and statistics as in Fig 3E. In (A-B) worms were grown on RNAi E. coli HT115 plates. (TIF) [file ppat.1008826.s005.tif]

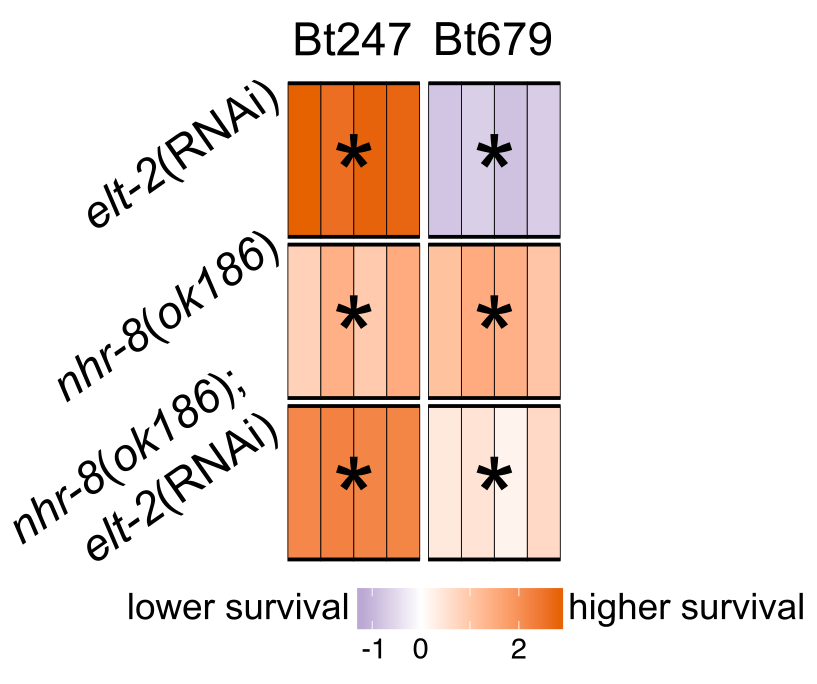

Supplement: S6 Fig — Difference in survival between the N2 control and the nuclear hormone receptor mutants nhr-8(ok186). A GLM of the binomial family was fitted followed by a Tukey HSD Test (see methods section), where mutant or knockdown worm strains were compared to control strains. Asterisks show significant differences between knockout/knockdown treatment and wildtype N2 control. p-value Bonferroni adjusted. Data represented in heatmap and statistics as in Fig 3E. Triangles indicate significant difference compared to elt-2(RNAi). (TIF) [file ppat.1008826.s006.tif]
