# Supplementary material for: The C. elegans GATA transcription factor elt-2 mediates distinct transcriptional responses and opposite infection outcomes towards different Bacillus thuringiensis strains
Source: PLoS Pathog. 2020 Sep 24;16(9):e1008826. doi: 10.1371/journal.ppat.1008826 (PMC7513999; doi:10.1371/journal.ppat.1008826)
Supplement: Attachment — Submitted filename: Zarate_Potes_et al_responses to reviewers.docx [file ppat.1008826.s016.docx]

Reviewer's Responses to Questions in grey

Our responses in blue

------------------------------------------------------------------------------

We thank the reviewers for their helpful comments, which we feel have allowed us to further improve the manuscript. We address the individual points raised by the reviewers and changes made in the manuscript below.

**Part I - Summary**
Please use this section to discuss strengths/weaknesses of study, novelty/significance, general execution and scholarship.

Reviewer #1: Zárate-Potes, et al. report a significant finding that differential host response to pathogen infection is dramatically influenced by gene expression responses to that infection. This finding is unexpected because as one might predict that loss of a key transcription factor required for normal host response would only render the host compromised for all pathogens. Rather, the authors report that reduction of elt-2 by RNAi renders C. elegans more resistant to one isolate and more sensitive to another. One particularly exciting finding is shown in Figure 4 where there was a change in intestinal integrity in response to different Bt isolates despite equivalent pathogen load. Although the mechanistic basis for this differential outcome remains unclear, the outcome is well documented and will certainly be valuable to the field and spark further studies.

Reviewer #2: This manuscript describes an extensive investigation of the contrasting effects of two Bacillus thuringiensis strains (Bt679 and Bt247) on the nematode Caenorhabditis elegans. Both are able to kill C. elegans, mainly or completely by intestinal damage caused by their different Cry toxins, but the authors show by transcriptome analyses that the responses of the worms have major differences. Exposure of worms to a toxin-negative Bt strain (Bt407) has similar transcriptomic consequences to exposure to standard E. coli food, which is reassuring, but the two toxic Bt strains elicit responses that indicate both shared and distinct consequences to the different strains. The importance of these effects was explored with appropriate mutants or RNAi knockdown. Major pathways implicated in the shared responses include the AP1 factor jun-1; the p38 MAP kinase pathway contributes to resistance to Bt679 but not to Bt247; and the ELT-2 transcription factor has opposite effects in the responses to the two strains.

The most striking result is that RNAi knockdown of the major intestinal transcription factor ELT-2 (a GATA factor) caused increased sensitivity to Bt679, as might be expected, but dramatically increased survival in the presence of Bt247. The authors show that the improved survival is probably due to increased tolerance, as opposed to resistance, because the intestinal damage and pathogen loads are apparently unchanged by elt-2(RNAi).

Much of the manuscript is devoted to attempts, mostly inconclusive, to explain this remarkable effect. The authors write, “increase [of elt-2(RNA worms] in survival on Bt247 is multifactorial, influenced by the nuclear hormone receptors NHR-99 and NHR-193, and also involves lipid metabolism, detoxification, and trehalose catabolism”, but most of this involvement is guesswork and speculation, unfortunately. The putative transcription factors NHR-99 and NHR-193 are shown to have an effect, but their targets are unknown. Analyzing the effects of ELT-2 is bound to be hugely complicated since it has transcriptional effects on at least one third of the C. elegans genome (e.g. page 28: 6180 genes are affected by elt-2(RNAi).

We agree with the reviewer that the effects of *elt-2* inactivation are complex as *elt-2* is a global regulator of intestine-expressed genes. However, our finding of the involvement of two so far uncharacterized NHRs, NHR-99 and NHR-193, is important, because it enhances our understanding of this remarkable defense phenotype and possibly the worm’s defense in general, even if the ligands are yet to be determined. Moreover, our transcriptome analysis of *elt-2*(RNAi) worms indicates an involvement of lipid metabolism and detoxification. To account for the reviewer’s comment and to avoid overselling, we now toned down our conclusions to emphasize that our results provide indications about the underlying mechanisms, which need to be validated in future studies. We now omitted carbohydrate and trehalose catabolism as we realized that we only discuss detoxification and lipid metabolism in the text.

Specifically, to respond to the reviewer’s concern we introduced the following changes:

- p. 3 (abstract) Changed: “We further demonstrate that the *elt-2* silencing-mediated increase in survival on Bt247 is multifactorial, influenced by the nuclear hormone receptors NHR-99 and NHR-193, and also involves lipid metabolism, detoxification, and trehalose catabolism” to “Our results further suggest that the *elt-2* silencing-mediated increase in survival on Bt247 is multifactorial, influenced by the nuclear hormone receptors NHR-99 and NHR-193, and may further involve lipid metabolism and detoxification.”

- p.35 (Final paragraph): Changed “.Gene expression analysis points to involvement of multiple candidate mechanisms such as detoxification, trehalose metabolism, and lipid metabolism.” to “Gene expression analysis points to a possible involvement of multiple candidate mechanisms such as detoxification and lipid metabolism, which need to be validated in future studies.”

Moreover, contributions of related/redundant GATA factors such as ELT-7 need to be considered.

We now discuss the three intestinal *C. elegans* GATA transcription factors (p. 16) and present results on two *elt-7* mutants (p.16, 17). The results were inconclusive as *elt-7*(*ok835*) and *elt-7*(*tm840*) mutants affected survival in opposite ways (Figure S1C). However, *elt-7* mutant animals do not exhibit a Bt strain-specific survival phenotype, while *elt-2*(RNAi) worms do, in consistency with the initial transcriptome data. These findings thus justify our focus on the GATA TF ELT-2.

Sterility effects are not worth reporting in such detail, or including in Figure 8 unless qualified with yet more question marks.

Our findings show that the increased survival rate of *elt-2*(RNAi) worms infected with Bt247 can be influenced by sterility and we thus included this finding in Figure 8. However, it is true that sterility does not show strain-specific effects, and we thus shortened the presentation of the results in the result section (p. 28) and removed sterility effects in the model for Bt247 presented in Figure 8, according to the reviewer’s suggestion.

The authors present some excellent EM data, and do a nice job of analyzing the positive contributions of ELT-2 and the p38 MAP kinase pathway to resistance.

**Part II – Major Issues: Key Experiments Required for Acceptance**
Please use this section to detail the key new experiments or modifications of existing experiments that should be absolutely required to validate study conclusions.

Generally, there should be no more than 3 such required experiments or major modifications for a "Major Revision" recommendation. If more than 3 experiments are necessary to validate the study conclusions, then you are encouraged to recommend "Reject".


Reviewer #1: 1. The major concern with this manuscript is the authors’ concluding model that depicts a scenario where elt-2 mediates two distinct responses to different pore-forming toxin inputs. Is it not more likely that intact elt-2 mediates one response program and loss of elt-2 function shows different outcomes depending on the insult? In other words, the different outcome is the result of how loss of elt-2 alters gene expression such that it primes animals beneficially for one toxin and renders animals susceptible to the other. The authors provide some evidence for their conclusion with the nhr versus pmk-1 results but this level of epistasis analysis is not conclusive enough to depict the two responses as entirely distinct as shown in the model. If the authors’ model is correct, it would be incumbent to demonstrate that elt-2 acts not only as a mediator of transcriptional programs in response to pathogens but also as a sensor of the specific pathogen.

We thank reviewer #1 for this important point. We now adjusted the model, taking into account the reviewer’s comments. In particular, we placed *elt-2* in the middle, in order to emphasize its central role in mediating distinct responses to the two different Bt strains, which we demonstrated by our finding of the differential regulation of the known ELT-2 targets by Bt247 and Bt679 exposure (clusters 2 and 8 in the first transcriptome data set, Figure 2B). This central role was further supported by disappearance of this strains-specific DE pattern upon *elt-2* knockdown (red boxes in Figure 7A). These aspects are now detailed in the figure legend. In the figure itself, we further point to other transcription factors such as NHRs that appear to interact with *elt-2* to hone the transcriptional response to a specific pathogen, as also explained in the figure legend. In the figure legend, we now further clarify that (i) the responses to Bt247 and Bt679 are not completely different, (ii) *elt-2* does not act as a direct sensor of a specific pathogen, but that the differential activation of the *elt-2* targets must be initiated by an upstream sensor, which still needs to be identified in future work.

2. Perhaps the weakest conclusion of this study is whether necrotic cell death is or is not involved in the pathogenesis. The authors make some contradictory statements about the role of necrosis. In the text, they state they did not find consistent responses on the two Bt isolates with loss of different factors in the necrotic pathway yet in the figure legend title they state that specific responses do not involve the necrosis pathway. I think this issue needs to be clarified. Are there any cellular morphological changes in response to the two distinct Bt isolates indicating similar or different necrotic cell death outcomes?

Again, we thank the reviewer for pointing out an ambiguity in our manuscript. We tested the involvement of the necrosis pathway in *elt-2*(RNAi)-mediated resistance to Bt247 infection, as the necrosis terminal aspartic protease gene *asp-1* was previously shown to mediate susceptibility to the PFT Cry6Aa (Zhang et al., 2016. PLOS Pathogens. doi:10.1371/journal.ppat.1005389). However, in the case of exposure to Bt247, the *asp-1* mutant was significantly more tolerant than control worms in only 1 out of five runs. We speak of inconsistent results as the *asp-1* mutant was significantly more susceptible in another run (out of these five runs) and thus showed the opposite phenotype. Our results thus do highlight that *asp-1* does not play a role as a susceptibility factor in Bt247 infection – in clear contrast to its role in the response to Cry6A exposure. In addition to *asp-1*, we tested two other necrosis pathway mutants, *itr-1* and *vha-12*. Here, our results are similarly inconsistent as *itr-1* mutants are more susceptible in three out of four runs, while *vha-12* mutants were more resistant in three out of four runs. The same mutants were clearly more resistant to Cry6A exposure (Zhang et al., 2016).

Since these mutants show contrasting survival phenotypes upon infection and since the *asp-1* mutant does (on average) not show a survival phenotype, we conclude that deficiency in necrosis is unlikely to have a strong effect on tolerance to Bt247. Because of this lack of evidence of a clear role of the necrosis pathway in tolerance to Bt247 infection, we did not further explore the role of necrosis during Bt247 infection. Investigating cellular morphological changes in response to the two Bt isolates, as suggested by the reviewer, would clearly go beyond the scope of this paper. We want to point out that reviewer #2 suggests to shorten or omit most of the results on p.25-28, including the results on the necrosis pathway. However, we now clarify our conclusion also in the text (p. 25, 26) and not only in the figure legend, as pointed out by the reviewer #1. We further explain our rationale to include variable results, following the arguments presented above.

Reviewer #2: 1. A general criticism of this work is that most of what has been looked at seems to be the consequences of intoxication rather than infection. This is especially true for Bt247, which does not appear to proliferate in the C. elegans intestine. This issue and its implications need to be better addressed.

We agree that infection implies the invasion and proliferation of pathogenic bacteria within the host and it is true that we only observed a multiplication of vegetative cells in living animals for Bt679. At the same time, we do not expose worms to purified toxins, but to Bt spores that are the natural infectious stage and these Bt spores do accumulate in the gut, which is consistent with the term ‘infection’, as commonly used in the literature. Moreover, we focused our morphological characterization of the infection process to living worms, for which we expect immune responses to be active. However, proliferation of vegetative cells occurs for both pathogens in dead worms, that is worms killed by either of the two pathogens, as we repeatedly demonstrated for these two strains in the past. In consideration of this context, ‘intoxication’ is also not the right term. To address the concern of the reviewer and to avoid any misunderstandings, we now explain our definition of the term ‘infection’ when it is used the first time (p.8 and p.9 first paragraph of results and discussion) and we included a picture of a Bt247-infected worm with spores in the intestinal lumen (Fig 1B).

2. The authors [showed] that purified toxins from Bt679 can kill, and showed that Cry21Aa3 rather than Cry14Aa2 is responsible, but purified Cry6Ba from Bt247 had no effect, although deletion of the relevant gene from Bt247 removed toxicity. This is disappointing and needs more explanation or investigation.

The fact that Cry6Ba alone is not responsible for toxicity (as opposed to Cry6Aa from another strain) is in our opinion not disappointing, but in contrast very interesting. Cry6Ba was shown to have coleoptericidal activity (Sharma et al. 2011. Curr. Microbiology doi:10.1007/s00284-010-9749-4.), but only limited nematocidal activity compared to Cry6Aa (Wei et al. 2003 PNAS doi:10.1073/pnas.0538072100). Also, Cry6 toxins are hemolysin E (HlyE)-like toxins (also known as Cytolysins A) and there are numerous examples of synergistic activities between cytolysins and other virulence factors (e.g., Pirie et al. 2013 doi:10.1158/1535-7163.MCT-12-1023; reviewed in Los et al. 2013 doi:10.1128/MMBR.00052-12). To address the reviewer’s concern, we now elaborate on these characteristics of Cry6Ba in the main text (p. 23, 2^nd^ paragraph).

3. Most of the experimentation on p.25-28 and in Figure 6 should be greatly shortened or omitted, as much of it is negative, inconclusive or inconsistent (as the authors admit).

We find it important that such variable results are openly presented and explained, even though this is usually not the case. Please note that our assay usually produces highly consistent results across truly independent biological replicates (see for example the results for *elt-2* RNAi). If in other cases, the results are variable and not consistently negative, then this does indicate that the respective gene can have at least a minor effect on the considered phenotype – in contrast to clear evidence for an absence of an effect. However, to address the concern of the reviewer we shortened the part on sterility, moved the results on the sterility mutants to the supplements, and also explained our rationale for highlighting variable results (following the arguments given above).

**Part III – Minor Issues: Editorial and Data Presentation Modifications**
Please use this section for editorial suggestions as well as relatively minor modifications of existing data that would enhance clarity.


Reviewer #1: 1. Figure 1 has a couple of issues that need to be amended. First, the worm image appears to be very similar to WormAtlas and that should be cited if true.

We thank the reviewer for pointing out these issues with figure 1. The worm image was produced by us but inspired by the illustrations found in WormAtlas, so we have included the corresponding citation preceded by “modified from” in the figure legend.

Second, the legend needs to be clarified to match what is being shown in A versus B.

We have included a new sentence in the figure legend of figure 1 explaining what is shown in panel (A). The explanations of panels (B-Q) follow in a separate sentence.

Third, the authors indicate that microvilli are artificially colored. Anatomically, it appears that the apical membrane is what has been marked throughout. Please clarify these issues in the legend.

We have marked the microvilli, to the best of our knowledge. The microvilli are a subdomain of the apical membrane and as they are best to be discerned on the TEM graphs (because they are membrane protrusions), we marked them in red. We have however changed the legend of Figures 1 and 4 to state that “The microvilli of the apical membrane are marked throughout and artificially colored in red to point out their integrity during infection.”, in response to the reviewer’s comment.

2. The title comes across as a bit ostentatious and uninformative. The data seem to show that elt-2 GATA factor regulates a transcriptional response that results in distinct outcomes depending on the pore toxin insult. The title should more adequately reflect those findings.

Following the reviewer’s comment, we adjusted the title to: ”**The *C. elegans* GATA transcription factor *elt-2* mediates distinct transcriptional responses and opposite infection outcomes towards different strains of the same pathogen species**”

3. A major conclusion of the authors is that the jun-1 transcription factor regulates the common response to the Bt strains whereas the elt-2 transcription factor mediates the strain-specific response. One could argue that the authors have found two transcriptional response pathways acting in parallel or the elt-2 is a subset of the jun-1. Thus it is unnecessary to distinguish one as regulatory and the other as a mediator given the evidence in hand. “Mediate” could be concluded for both without diminishing the impact of the finding.

We went through the text and changed “regulate” to “mediate” in the abstract according to the reviewer’s suggestion.

- p. 3 (abstract): Changed “*jun-1* regulates” to “*jun-1* mediates”

Reviewer #2: 4. Page 15 Figure 3G is either unnecessary or wrong.

We thank the reviewer for pointing out this issue with Figure 3G. We have now moved it to Figure S1B.

In the figure legend of Figure S1 we explain that commonly Bt679 is more virulent than Bt247, requiring a lower concentration of spores to produce the same extent of host killing as Bt247 (what we now illustrate in Figure S1A). However, in one occasion we obtained a Bt247 culture which had the same level of virulence as the Bt679 culture, meaning that the same concentration of spores for both Bt247 and Bt679 produced the same extent of host killing. With figure S1B we want to show that even when both strains have the same level of virulence the effect of *elt-2*(RNAi) on survival rate remains unchanged, indicating that the effect does not depend on the differences in virulence between these two strains.

To further clarify this, we changed “The strain Bt407 served as a non-pathogenic, non-Cry PFT-producing control. The more pathogenic Bt679 produces the nematocidal 3d-Cry PFTs Cry21Aa3 (Cry21Aa) and Cry14Aa2 (Cry14Aa), while the less pathogenic Bt247 only expresses a unique, structurally distinct toxin, Cry6Ba” in the introduction (p. 8, first paragraph) to “The strain Bt407 served as a non-pathogenic, non-Cry PFT-producing control. The generally more virulent Bt679 produces the nematocidal 3d-Cry PFTs Cry21Aa3 (Cry21Aa) and Cry14Aa2 (Cry14Aa), while the generally less virulent Bt247 only expresses a unique, structurally distinct toxin, Cry6Ba”.

5. p.22 line -7 ‘after Bt247 infection’ should be presumably be ‘after Bt679 infection’.

We have rewritten the text of the results and discussion section in page 22 to better explain our results of the epistasis analysis of the p38 MAPK pathway and *elt-2* after Bt247 and Bt679 infection. The text now reads as follows: “*pmk-1*(*km25*);*elt-2*(RNAi) animals displayed a higher survival rate compared to *elt-2*(RNAi) and control animals after Bt247 infection (Figure 5A, Figure S3A). *pmk-1*(*km25*);*elt-2*(RNAi) animals were, however, significantly more susceptible than *elt-2*(RNAi) and *pmk-1*(*km25*) mutant worms alone after Bt679 infection, indicating that *elt-2* acts in parallel to the p38 MAPK pathway in mediating resistance to Bt679 (Figure 5A, Figure S3B), possibly by regulating the expression of complementary sets of immune effector genes.”

Also, the Bt247 data in Figure 5A have triangles indicating a stronger effect in elt-2(RNAi); pmk-1 than in elt-2(RNAi) alone, but the heatmaps look identical.

We thank the reviewer for pointing out this problem with our heatmaps, which makes it difficult in particular cases to distinguish subtle but significant differences between treatment groups. We have re-assessed the raw data used to construct the heatmap in figure 5A (also supplied in Table S8 and results of the statistical tests supplied in Table S9) and can confirm that indeed the survival rates of *pmk-1*(*km25*);*elt-2*(RNAi) worms after Bt247 infection are significantly higher than the survival rates of *elt-2*(RNAi) worms, despite the difference between them being quantitatively very small, leading to only a slight difference in the coloration of the heatmap. We have now included these data in Figure S3A plotted as survival curves, where the difference in survival rate between *pmk-1*(*km25*);*elt-2*(RNAi) and *elt-2*(RNAi) worms after Bt247 infection is represented in a clearer way.

6. p.32 line -6 NHR-93 should be NHR-193.

NHR-93 has been changed to NHR-193. We thank the reviewer for pointing this out..

7. p. 30, 31 and throughout: Transcriptional reporters are preferably written as nhr-193p::GFP, not pnhr-193::GFP

The nomenclature of the transcriptional reporters has been changed throughout the text according to the reviewer’s comment.

8. p. 41 Zeiss Zeiss

We removed the repeated word.
